# How should the high-risk children go back to school following school reopening in the COVID-19 pandemic?

**DOI:** 10.17179/excli2021-3507

**Published:** 2021-02-26

**Authors:** Mehrdad Askarian, Mohammad Hossein Taghrir, Hossein Akbarialiabad

**Affiliations:** 1 Department of Community Medicine, School of Medicine, Shiraz University of Medical Sciences, Shiraz, Iran; Health Behavior Science Research Center, Shiraz University of Medical Sciences, Shiraz, Iran; 2 Department of Community Medicine, School of Medicine, Shiraz University of Medical Sciences, Shiraz, Iran; 3 Student Research Committee, Shiraz Medical School, Shiraz University of Medical Sciences, Shiraz, Iran

## ⁯⁯

***Dear editor,***

The school closure has been implemented in response to the COVID-19 pandemic; meanwhile, the process of school reopening has been a consistent solicitude for healthcare and educational policymakers to protect the students, teachers, and staff. The need to reopen schools is fundamental, and learning of social and emotional skills and competencies in the context of in-person learning is not negligible, especially for children. 

As far as school reopening is concerned, it is also crucial to determine the virulence of SARS-CoV-2 among school-aged children and how it can be transmitted in child-child, child-adult, and adult-child manner. An overview of recent studies from the United States, Israel, Netherlands, Iceland, Norway, Spain, Italy, Singapore, and France demonstrated that the attack rate among children is significantly lower compared with adults, and children are less possible the disease reservoir (Munro and Faust, 2020[6]). 

A recently published systematic review and meta-analysis was concerning this issue (Badal et al., 2021[1]). In this paper, twenty studies with 1810 cases were enrolled; 84 % were categorized into non-severe, and only 5 % presented a severe illness. In a report of 100 pediatric cases from Pediatric Emergency Departments in Italy, only two of 27 patients with a coexisting condition such as cystic fibrosis, neurological and hematological problems had a severe and critical illness (Parri et al., 2020[7]). In another study of 2133 pediatric patients from China, 125 (5,8 %) cases were defined as severe and critical (Dong et al., 2020[4]). In the aforementioned systematic review and meta-analysis, authors implicitly stated that those children who experienced severe COVID-19 were those who had “prior health condition.” Notably, in this comprehensive study, the types of comorbid conditions were not explicitly established; and this fact seems to be vaguely addressed in the literature, requiring more rigorous studies. 

If we comprehended that 23 % of pediatric confirmed cases were found to have a comorbid condition in a study, and 77 % of patients with known hospitalization status had comorbidity (CDC COVID-19 Response Team, 2020[2]), the story would sound dissonant. A multinational study from Europe reported 582 children with laboratory-confirmed COVID-19 (Götzinger et al., 2020[5]). Of those, 48 (8 %) required ICU admission; 25 % of total cases had pre-existing medical maladies such as chronic pulmonary disease, malignancy and were on immunosuppressive therapy, in comparison to 52 % of ICU admissions.

Based on what is mentioned above, children’s role in transmitting the disease is controversial, so it is rational to end the school closure. Also, by inferring from prior mentioned studies on hospitalized pediatric COVID-19 patients, we can affirm that most of them had an underlying disease and those with pre-existing medical problems developed more severe and critical presentations; the fact that Centers for Disease Control and Prevention (CDC) ratifies (CDC, 2020[3]). We believe that the risk of school reopening to children with comorbidities should be addressed more to warrant their safety, and global attention needs to focus on the aforementioned group.

Based on up-to-date evidence, children with the following conditions might be at amplified risk for severe illness: obesity, medical complexity, severe genetic disorders, severe neurologic disorders, inherited metabolic disorders, sickle cell disease, congenital heart disease, diabetes, chronic kidney disease, asthma and other chronic lung diseases, immunosuppression due to malignancy and those on immunosuppressive medical regimen (CDC, 2020[3]). Therefore, the following recommendations would be of benefits for these groups amid in-person school attendance.

- Create a process to convince high-risk students to identify themselves as susceptible individuals to COVID-19. The teacher and staff should educate all children and families (whether their child has comorbidity or not). Such psychological interventions would reduce the stigma and greatly diminish the inflicted children's mental health burden.
- Sign and symptoms of COVID-19 may be different in high-risk students. Screening programs should be aware of this.
- Have a plan to consider these students’ requests for alternative learning arrangements, including blended learning and other e-learning possibilities where applicable.
- Implement the infrastructures of other learning options, such as remote learning (providing high-speed internet access, digital equipment, and required resources). This statement is also valid about more innovative and remote assessment options. We believe that using novel assessment options such as portfolios may also reduce routine examinations' anxiety and stress. Meanwhile, the students can take advantage of the home time to work and improve their portfolios.
- Provide proper air conditioning in the classrooms and place high-risk students' seats in a well-ventilated place, such as near a window.
- Encourage the high-risk students to wear face-covering masks and face shields together to provide further protection. We highlight this point for only high-risk students as it may not be feasible for other students to follow such precautions.

## Funding

None.

## Conflict of interest

None.

## Authors’ contribution

All authors developed the idea and contributed to the final version of the manuscript equally.

## References

[1] Badal S, Thapa Bajgain K, Badal S, Thapa R, Bajgain BB, Santana MJ (2021). Prevalence, clinical characteristics, and outcomes of pediatric COVID-19: A systematic review and meta-analysis. J Clin Virol.

[2] CDC (2020). COVID-19 Response Team. Coronavirus Disease 2019 in Children - United States, February 12-April 2, 2020. MMWR Morb Mortal Wkly Rep.

[3] CDC (2020). People with certain medical conditions. https://www.cdc.gov/coronavirus/2019-ncov/need-extra-precautions/people-with-medical-conditions.html?CDC_AA_refVal=https%3A%2F%2Fwww.cdc.gov%2Fcoronavirus%2F2019-ncov%2Fneed-extra-precautions%2Fgroups-at-higher-risk.html.

[4] Dong Y, Mo X, Hu Y, Qi X, Jiang F, Jiang Z (2020). Epidemiology of COVID-19 among children in China. Pediatrics.

[5] Götzinger F, Santiago-García B, Noguera-Julián A, Lanaspa M, Lancella L, Carducci FIC (2020). COVID-19 in children and adolescents in Europe: A multinational, multicentre cohort study. Lancet Child Adolesc Health.

[6] Munro APS, Faust SN (2020). Children are not COVID-19 super spreaders: Time to go back to school. Arch Dis Child.

[7] Parri N, Lenge M, Buonsenso D, Coronavirus Infection in Pediatric Emergency Departments (CONFIDENCE) Research Group (2020). Children with Covid-19 in pediatric emergency departments in Italy. N Engl J Med.

